# Super-Hydrophobic/Icephobic Coatings Based on Silica Nanoparticles Modified by Self-Assembled Monolayers

**DOI:** 10.3390/nano6120232

**Published:** 2016-12-02

**Authors:** Junpeng Liu, Zaid A. Janjua, Martin Roe, Fang Xu, Barbara Turnbull, Kwing-So Choi, Xianghui Hou

**Affiliations:** Faculty of Engineering, University of Nottingham, University Park, Nottingham NG7 2RD, UK; junpeng.liu@nottingham.ac.uk (J.L.); evxzj3@exmail.nottingham.ac.uk (Z.A.J.); martin.roe@nottingham.ac.uk (M.R.); fang.xu@nottingham.ac.uk (F.X.); barbara.turnbull@nottingham.ac.uk (B.T.); kwing-so.choi@nottingham.ac.uk (K.-S.C.)

**Keywords:** super-hydrophobic, icephobic, silica nanoparticles, fluorosilane, self-assembled monolayers, durability

## Abstract

A super-hydrophobic surface has been obtained from nanocomposite materials based on silica nanoparticles and self-assembled monolayers of 1*H*,1*H*,2*H*,2*H*-perfluorooctyltriethoxysilane (POTS) using spin coating and chemical vapor deposition methods. Scanning electron microscope images reveal the porous structure of the silica nanoparticles, which can trap small-scale air pockets. An average water contact angle of 163° and bouncing off of incoming water droplets suggest that a super-hydrophobic surface has been obtained based on the silica nanoparticles and POTS coating. The monitored water droplet icing test results show that icing is significantly delayed by silica-based nano-coatings compared with bare substrates and commercial icephobic products. Ice adhesion test results show that the ice adhesion strength is reduced remarkably by silica-based nano-coatings. The bouncing phenomenon of water droplets, the icing delay performance and the lower ice adhesion strength suggest that the super-hydrophobic coatings based on a combination of silica and POTS also show icephobicity. An erosion test rig based on pressurized pneumatic water impinging impact was used to evaluate the durability of the super-hydrophobic/icephobic coatings. The results show that durable coatings have been obtained, although improvement will be needed in future work aiming for applications in aerospace.

## 1. Introduction

Ice formation and accretion may hinder the economic and environmentally friendly operation of aircraft [1] and pose a serious hazard that may cause accidents. For aircraft, it is necessary to have a de-icing and anti-icing system on the ground and during flight. However, current de-icing and anti-icing systems release chemicals into the environment, build up weight, increase fuel consumption and add complexity to the aircraft systems [2]. Aiming for an environmentally friendly and cost-effective way to solve the issue of ice formation and accretion, a durable icephobic coating on the surface of aircraft is potentially an ideal solution.

A surface that exhibits a water contact angle of 150° or greater with very little flow resistance is considered to be super-hydrophobic [3]. Super-hydrophobic surfaces are effective in allowing the incoming water droplets to bounce off, delaying ice formation and reducing the ice adhesion strength [4]. In order to fabricate super-hydrophobic surfaces, both the surface chemical composition and morphology need to be tuned to achieve a low surface energy and desirable surface roughness [5]. Various methods have been developed to prepare a rough surface from a low-surface-energy material or to modify a rough surface with a low-surface-energy material, such as electrochemistry, mechanical machining, chemical etching, spin coating and chemical vapor deposition [6,7,8,9,10,11,12]. Among them, a combination of spin coating of a rough material and chemical vapor deposition of a low-surface-energy material is straightforward and inexpensive.

Coatings incorporating silica nanoparticles have been attracting significant interest due to high thermal and mechanical stability and high surface roughness [13]. Among low-surface-energy materials, fluoroalkyl silanes are promising for practical applications because of their high mechanical and chemical stability resulting from strong immobilization through siloxane bonding [14]. In previous research, hydrophobic coatings based on silica were widely reported. However, the icephobicity, icing behavior and durability of coatings based on silica nanoparticles were less investigated. In addition, the durability of hydrophobic/icephobic coatings is very important for practical applications, especially in aircraft applications, and has remained challenging. Xu et al. [15] reported an erosion test method based on the impingement of water droplets released from a higher stage using gravity. In this experiment, an erosion test rig with the impact of impinging by high-velocity pneumatic water was set up and used to evaluate the durability.

In the current work, silica nanoparticles were deposited by the spin-coating method to form a nanostructured rough surface to trap small-scale air pockets. Self-assembled monolayers (SAMs) of fluoroalkyl silane, 1*H*,1*H*,2*H*,2*H*-perfluorooctyltriethoxysilane (POTS), were grafted onto the silica nanoparticle surface by the chemical vapor deposition method to obtain a low surface energy. The hydrophobicity, icephobicity and durability of the coatings were investigated.

## 2. Experimental Section

### 2.1. Fabrication of Silica-Based Nano-Coatings with Self-Assembled Monolayers

Silica nanoparticles, polystyrene and POTS (98%) were purchased from Sigma-Aldrich Company (Dorset, UK). Chloroform was purchased from Fisher Scientific Company (Loughborough, UK). All chemicals were used as received. 0.5 g silica nanoparticles and 0.019 g polystyrene were dissolved into 30 mL chloroform by continuous stirring for about 1 h. The mixture was deposited onto substrates at a speed of 1500 rad/min for 1 min using a spin coater (KW-4A, Chemat Group, Northridge, CA, USA). For ice adhesion test, the Al substrates with roughness average (*R*_a_) of 2.64 nm in area of 5 µm × 5 µm are alloy (2024-T4). For all other tests, the substrates are glasses with *R*_a_ of 1.66 nm in area of 5 µm × 5 µm.

Then the samples were transferred into a furnace for heat treatment at 550 °C for 2 h to remove the organic components and fuse the silica nanoparticles together. Then the silica based coatings with thickness of about 30 µm were formed. To reduce the surface energy and obtain super-hydrophobic surfaces, the samples were grafted by self-assembled monolayers of POTS using chemical vapor deposition method in a sealed vessel with 0.3 mL POTS at 180 °C for 3 h. Coatings based on commercial super-hydrophobic and icephobic silicone were also fabricated for comparison.

### 2.2. Characterization of Morphology, Composition and Hydrophobicity

The surface morphology was investigated by a scanning electron microscope (SEM, XL30, Philips FEI, Eindhoven, Netherlands) under an acceleration voltage of 20 kV after Pt was deposited on the samples to prevent charging by electron beam. The composition was measured by energy dispersive X-ray spectroscopy (EDS, Oxford Instruments plc., Oxfordshire, UK) with an electron accelerating voltage of 20 kV by accumulating the counts for 60 s. The binding energies of elements were characterized by an X-ray photoelectron spectroscopy (XPS, ESCALAB Mark II, VG Scientific, Waltham, MA, USA) using Al Kα X-ray as the radiation source with wavelength of 1486.6 eV. The Fourier transform infrared (FTIR) spectra were recorded by a spectrometer (Spectrum One, PerKin Elmer, Akron, OH, USA) using attenuated total reflection mode in the range between 650 cm^−1^ and 1300 cm^−1^. Hydrophobicity of the surfaces was characterized using a contact angle goniometer (FTA200, First Ten Angstroms, Inc., Portsmouth, VA, USA) with pumping out rate of 1 µL/s.

### 2.3. Icephobicity Test

Ice adhesion tests were performed using a centrifuge method with a glaze ice block (mass of 1.3 g) in a low temperature chamber with temperature of −5 °C. Using the rotation speed at the detachment of the glaze ice block, the ice adhesion strength is calculated using the ice block mass and beam length [16]:

(1) $$F=mr{ɷ}^{2}$$

where *F* is the centrifugal force (N), *m* is the mass of ice block (kg), *r* is the radius of the beam (m) and ɷ is the speed of rotation (rad/s). From the centrifugal force, the shear stress is determined:

(2) $$\tau =\frac{F}{A}$$

where *A* is the Area iced (m^2^), τ is the shear stress (Pa). Six silica-based coating samples were measured for better accuracy.

The water droplet icing tests were performed by monitoring the water droplets on three spots of coated samples and uncoated samples on a cold plate setting at −10 °C. By observing the video of the water droplets, icing duration can be obtained.

### 2.4. Durability Test

**Figure 1:**
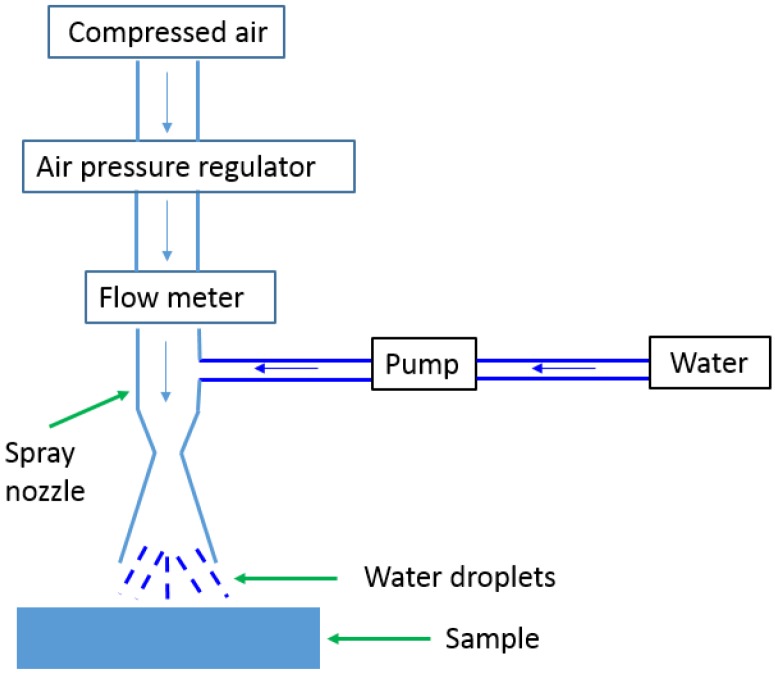
A schematic diagram of the water impinging test.

To evaluate the durability, erosion test rig (as shown in Figure 1) under pressurized pneumatic water impinging with gas pressure of 15 psi, velocity of 22 m/s and liquid flow rate of 22 mL/min was set up. Pressurized water droplets were spray onto the coated samples for various durations between 30 and 60 min. The water contact angle was measured on three spots before and after the erosion test.

## 3. Results and Discussion

### 3.1. Surface Treatment and Morphology

**Figure 2:**
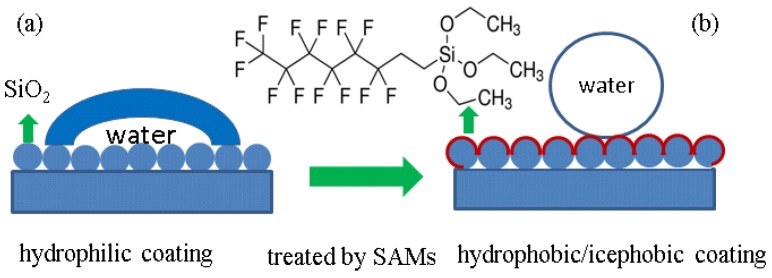
The schematic of the surface modification process by self-assembled monolayers and conversion from hydrophilic (**a**) to super-hydrophobic (**b**).

Figure 2 shows the schematic of the surface modification process and the conversion from hydrophilic to super-hydrophobic. Aiming to obtain a super-hydrophobic surface, silica nanoparticles were spin-coated onto the glass substrates, followed by the chemical vapor deposition of self-assembled monolayers of POTS to form a low surface energy on rough surfaces and covalent bonding between self-assembled monolayers of POTS and silica nanoparticles.

**Figure 3:**
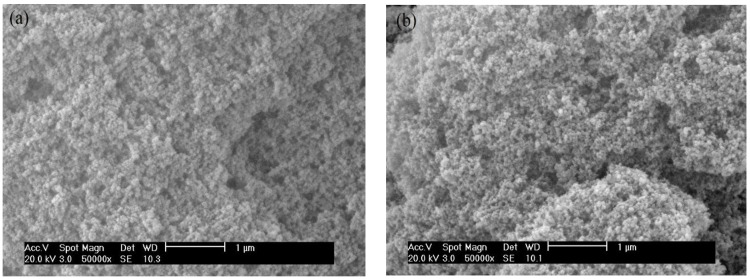
Scanning electron microscope (SEM) images of silica nanoparticles coating before (**a**) and after surface treatment (**b**).

The uniformity and morphology of the coatings before and after surface treatment were characterized by SEM techniques and the SEM images are shown in Figure 3. The images show distinguishable particles and porous structures which will allow the trapping of small-scale air pockets and reduce the fractional coverage at the solid-liquid interface. It can also be seen that the morphology is quite similar before and after treatment. There is no obvious change in the morphology of silica particles during the surface treatment as the POTS tends to be very thin self-assembled monolayers.

### 3.2. Confirmation of Self-Assembled Monolayers

**Figure 4:**
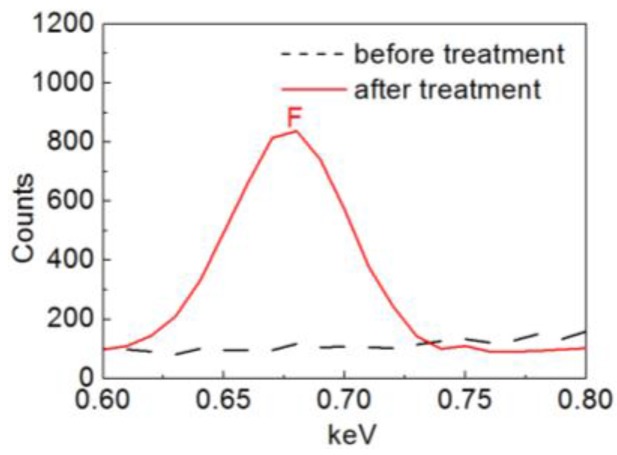
Energy dispersive X-ray spectroscopy (EDS) results for silica nanoparticles with fluoroalkyl silane, 1*H*,1*H*,2*H*,2*H*-perfluorooctyltriethoxysilane (POTS) treatment and without treatment.

To confirm whether the POTS had been successfully deposited onto the silica nanoparticles, elemental analysis was performed using EDS. There are five elements including H, C, F, O and Si in the structure of POTS. F is the best and most unique element to prove the existence of such POTS coatings because H is not easy to detect by EDS and any C detected may be a result of contamination. From Figure 4, it can be seen that there is a clear F peak after POTS treatment, while there is no F peak before POTS treatment. The EDS results therefore suggest that a POTS coating had been deposited onto the silica nanoparticles.

**Figure 5:**
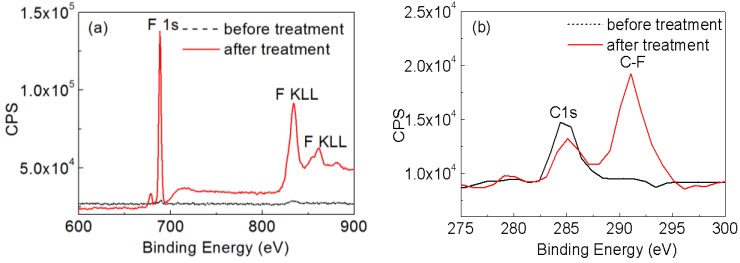
X-ray photoelectron spectroscopy (XPS) results for F (**a**) and C−F (**b**) of silica nanoparticles with treatment and without treatment by self-assembled monolayers of POTS.

To further verify the surface status and absorption of POTS of the treated silica particles and those before treatment, XPS analysis was carried out. Figure 5 shows the XPS results of the F1s, F KLL (the energy of the electrons ejected from the atoms due to the filling of the F1s state (K shell) by an electron from the L shell coupled with the ejection of an electron from an L shell), C1s and C−F regions of the spectra of the samples before and after treatment. From Figure 5a, it can be clearly seen that there is a F1s peak centered at 688.08 eV and F KLL peaks centered at 834.08 and 861.08 eV for the coating based on silica nanoparticles after POTS coating, while there is no F peak for the coating based on untreated silica nanoparticles. In the high-resolution scan for the C−F peak shown in Figure 5b, the C−F peak centered at 291.08 eV appears after POTS treatment while there is no C−F peak before treatment. The combined results of EDS and XPS confirm that self-assembled monolayers of POTS have been successfully grafted onto the silica nanoparticles. This is in good agreement with the previous results by Lai and Zhang et al. [14,17].

Understanding the formation mechanism of the SAMs is important for further optimization. In a previous report, it is inferred that the reaction starts from the hydrolysis of the POTS precursor which forms Si–OH bonds from the Si–OCH_2_CH_3_ bonds. Then, covalent linkage occurs through interfacial condensation and polymerization reactions between the hydroxyl groups and the silanol groups [14].

**Figure 6:**
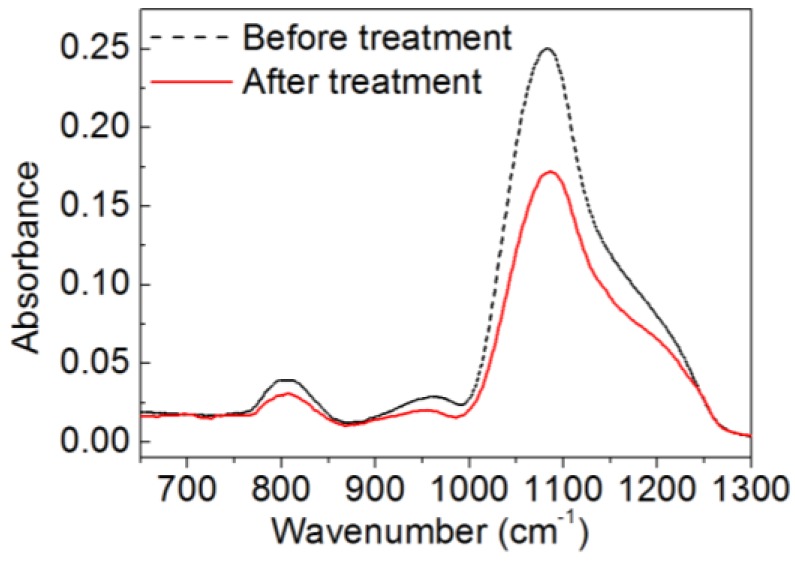
Fourier transform infrared (FTIR) absorption spectra of silica nanoparticles before and after treatment.

In the FTIR spectra shown in Figure 6, besides silica absorption peaks at about 810 cm^−1^ and 1086 cm^−1^, a Si–OH absorption peak around 965 cm^−1^ is observed from the samples before and after treatment [18,19,20]. The FTIR results suggest that the surface of the silica nanoparticles is terminated with –OH groups [21,22] which act as anchoring points for the formation of covalent bonds with the hydrolyzed POTS [6].

### 3.3. Surface Hydrophobicity

**Figure 7:**
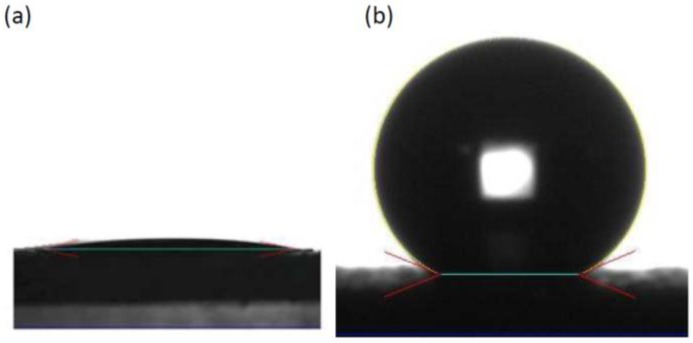
Water contact angle of water droplets on silica nanoparticles–based coating without (**a**) and with (**b**) POTS treatment.

The self-assembled monolayer of 1*H*,1*H*,2*H*,2*H*-perfluorooctyltriethoxysilane (POTS) will form low-surface-energy surfaces which will contribute to the super-hydrophobicity. Figure 7 shows the water contact angle of the water droplets on the silica coating without (Figure 7a) and with (Figure 7b) POTS treatment. The water contact angle changes from 13° ± 0.9° without treatment to 163° ± 7.4° measured for six samples after treatment with the same processing conditions, indicating a transition from hydrophilic to super-hydrophobic as a result of POTS treatment. The water droplets will bounce off from the surface in the case of a very small angle inclination of the sample surface, even if the angle of inclination is invisible. The bouncing off phenomenon of the water droplets is shown in Video S1.

The Wenzel model and the Cassie-Baxter model are generally used to explain the hydrophobicity of coatings with high roughness. In the Wenzel model, water droplets follow the profile of a rough surface and are pinned to the surface, which results in them being unable to slide on the surface [18]. However, in the silica nanoparticles–based samples, water droplets tend to slide on the surface very easily, suggesting the Cassie-Baxter model is more suitable to explain our experimental results. In Cassie’s equation:

cosθ_A_ = *f*_1_cosθ − *f*_2_, (3)

where θ_A_ is the apparent contact angle measured on the substrate surface; θ is the water contact angle on the fluoridated smooth surface and it was 100° [19]; *f*_1_ and *f*_2_ are the fractions of the solid surface and air in contact with water droplets; and *f*_1_ + *f*_2_ = 1 [20]. The *f*_1_ calculated using the average water contact angle of 163° is 5.3% and it indicates that 94.7% of the surface is occupied by air, which indicates that a combination of silica nanoparticles and POTS allows air to be trapped easily, resulting in a super-hydrophobic surface.

### 3.4. Water Droplet Icing Behavior

**Figure 8:**
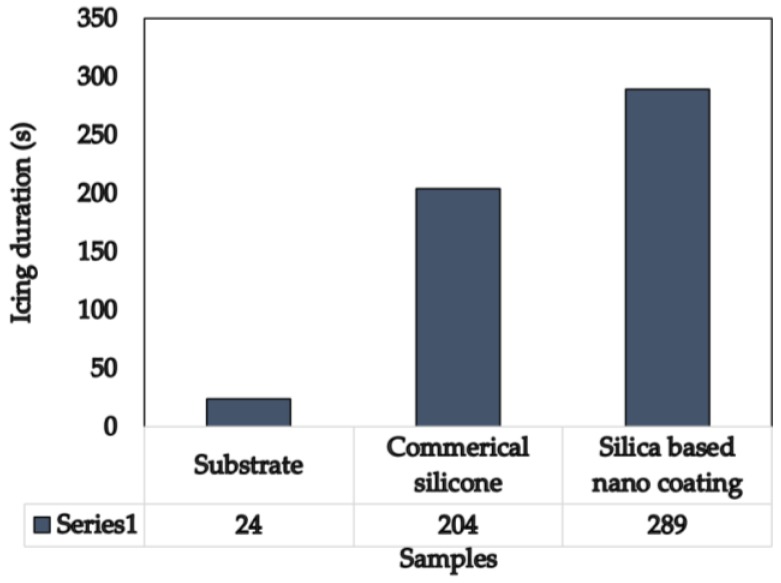
Water droplet icing test results.

According to classical nucleation theory and observation, it was reported that the nucleation rate and macroscopical growth velocity of ice can be greatly reduced by a super-hydrophobic surface owing to an extremely low, actual solid-liquid contact area caused by the trapped air pockets [4]. As previously discussed, the reduced solid-liquid interface fraction of 5.3% will contribute to an icing delay due to the limited thermal exchange between the solid-liquid surface. The water droplet icing test results in Figure 8 show that 289 s were needed for the formation of ice on the super-hydrophobic surface of the silica-based nano-coating and 24 s were needed for the bare substrates. For the commercial silicone icephobic samples, 204 s were needed for ice formation. The water droplet icing test results of coated samples show a significant delay in icing compared with the bare substrates and an improvement in icephobicity compared with the commercial icephobic products.

### 3.5. Ice Adhesion Strength

**Figure 9:**
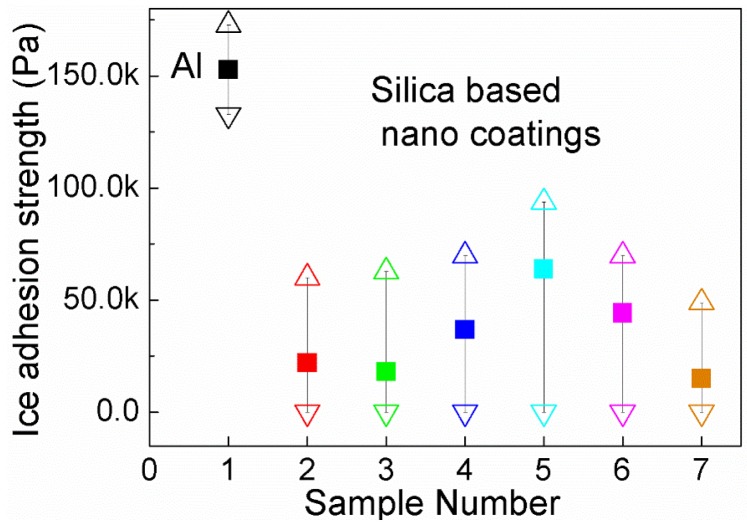
Ice adhesion results of silica-based nano-coatings on Al substrates (samples 2–7) and untreated Al surface (sample 1).

Besides the icing delay performance, the ice adhesion strength is another important parameter for icephobicity. With low ice adhesion strength, the ice can be removed easily which is desirable for de-icing. It was revealed that the average ice adhesion strength is linearly correlated with 1 + cosθ_e_, with θ_e_ standing for the estimated equilibrium contact angle which implies that a low ice adhesion strength can be obtained from super-hydrophobic surfaces [1]. In this experiment, the centrifuge adhesion test method was used to evaluate the ice adhesion strength of silica-based nano-coatings and aluminium substrates for comparison [16]. From Figure 9, it can be seen that all the measured shear stresses between the coated samples/glaze ice block were remarkably less than the shear stresses between the Al substrates/glaze ice block. There are some variations in the ice adhesion results. For better accuracy, we tested six silica-based coating samples fabricated by the same formulation. The difference between each sample, especially between samples 5 and 7, might be caused by natural variability in the shapes of ice blocks. The shear stresses between the ice and the silica-coated samples are all lower than 100 kPa which is the threshold for icephobicity [21]. It is worth mentioning that some glaze ice blocks were dropped from the silica-coated sample before the rotation started showing extremely low ice adhesion strength.

A strict definition of icephobicity remains unclear. It was suggested that a surface should be called icephobic if it delays ice formation at temperatures below the freezing point of water and/or if it has a weak adhesion strength to ice of less than 100 kPa [21]. The bouncing off of incoming water droplets, the icing delay performance and the low ice adhesion strength show that the super-hydrophobic coatings based on silica nanoparticles are suitable for use as icephobic coatings regarding their icephobicity.

### 3.6. Durability under Impact of Pneumatic Water Impinging

**Figure 10:**
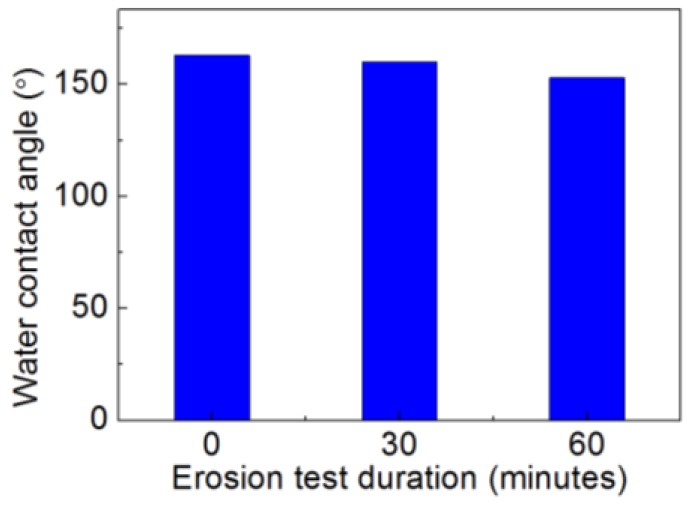
Water contact angle before and after erosion test from water impinging for silica-based nano-coatings for as-prepared sample, after 30 min test and after 60 min test.

When an aircraft flies through the atmosphere, its surfaces may undergo impact from hydrometeors such as rain, which can adversely affect the structure of the aircraft and reduce the lifecycle of the components [22]. Therefore, the durability performance of the hydrophobic/icephobic coatings is a critical factor for practical applications in aircrafts. In this experiment, pneumatic water impinging was used in the erosion test rig to evaluate the durability. Figure 10 shows the water impinging test results for silica-based coatings for the as-prepared sample, after a 30 min test and after a 60 min test. The super-hydrophobicity remained after the erosion test for 60 min. Although the water contact angle dropped from 163° ± 7.4° to 161° ± 4.9° after the 30 min erosion test and to 153° ± 2.6° after the 60 min test, the degeneration of hydrophobicity is at a reasonable value, indicating a certain durability. However, aiming for applications in aerospace, further optimization will be performed to improve the durability.

## 4. Conclusions

Silica nanoparticles were deposited onto glass substrates to form a nanostructured rough surface with the function of trapping small-scale air pockets. Self-assembled monolayers (SAMs) of 1*H*,1*H*,2*H*,2*H*-perfluorooctyltriethoxysilane were grafted onto the silica nanoparticle surface by the chemical vapor deposition method to reduce the surface energy. The morphology, composition, and functional groups were characterized to reveal the relationship between the characteristics of the nanocomposite material and the hydrophobicity. An average water contact angle of 163° suggests a super-hydrophobic surface was obtained on silica nanoparticles with surface modification by SAMs of POTS. The water droplet icing test results show that the icing formation of silica-based nano-coatings was significantly delayed compared to bare substrates and commercial icephobic products due to the existence of the low surface energy and air pockets on the surface. The ice adhesion strength test results show that the shear stresses between the treated surface/ice block are much lower than those between the bare substrate/ice block. The icing delay and low ice adhesion strength suggest icephobic surfaces have been obtained from the super-hydrophobic silica-based coatings. To evaluate durability, a test rig of erosion from pneumatic water impinging was designed and set up. The erosion test results show that super-hydrophobicity remained after testing for 60 min. Further optimization aiming for aircraft applications is in progress.

## Supplementary Materials

The following are available online at http://www.mdpi.com/2079-4991/6/12/232/s1, Video S1: The water droplets bouncing off phenomenon.

## References

[1] Meuler A.J., Smith J.D., Varanasi K.K., Mabry J.M., McKinley G.H., Cohen R.E. (2010). Relationships between water wettability and ice adhesion. ACS Appl. Mater. Interfaces.

[2] Gent R., Dart N., Cansdale J. (2000). Aircraft icing. Philos. Trans. R. Soc. Lond. A.

[3] Karunakaran R.G., Lu C.-H., Zhang Z., Yang S. (2011). Highly transparent superhydrophobic surfaces from the coassembly of nanoparticles (≤100 nm). Langmuir.

[4] Shen Y., Tao J., Tao H., Chen S., Pan L., Wang T. (2015). Anti-icing potential of superhydrophobic Ti_6_Al_4_V surfaces: Ice nucleation and growth. Langmuir.

[5] Zhang X., Shi F., Niu J., Jiang Y.G., Wang Z.Q. (2008). Superhydrophobic surfaces: From structural control to functional application. J. Mater. Chem..

[6] Wang N., Xiong D., Deng Y., Shi Y., Wang K. (2015). Mechanically robust superhydrophobic steel surface with anti-icing, uv-durability, and corrosion resistance properties. ACS Appl. Mater. Interfaces.

[7] Chane-Ching K.I., Lacroix J.C., Jouini M., Lacaze P.C. (1999). Electropolymerization of hydrophobic dipyrrolyls in aqueous medium based on inclusion chemistry. J. Mater. Chem..

[8] Chu D., Nemoto A., Ito H. (2014). Hydrophobic property of hierarchical polymer surfaces fabricated by precision tooling machine. J. Polym. Eng..

[9] Esmaeilirad A., Rukosuyev M.V., Jun M.B.G., van Veggel F. (2016). A cost-effective method to create physically and thermally stable and storable super-hydrophobic aluminum alloy surfaces. Surf. Coat. Technol..

[10] Tien H.W., Huang Y.L., Yang S.Y., Hsiao S.T., Liao W.H., Li H.M., Wang Y.S., Wang J.Y., Ma C.C.M. (2012). Preparation of transparent, conductive films by graphene nanosheet deposition on hydrophilic or hydrophobic surfaces through control of the ph value. J. Mater. Chem..

[11] Ponja S., Sathasivam S., Chadwick N., Kafizas A., Bawaked S.M., Obaid A.Y., Al-Thabaiti S., Basahel S.N., Parkin I.P., Carmalt C.J. (2013). Aerosol assisted chemical vapour deposition of hydrophobic TiO_2_-SnO_2_ composite film with novel microstructure and enhanced photocatalytic activity. J. Mater. Chem. A.

[12] Lin C.C., Hsu S.H., Chang Y.L., Su W.F. (2010). Transparent hydrophobic durable low moisture permeation poly(fluoroimide acrylate)/SiO_2_ nanocomposite from solventless photocurable resin system. J. Mater. Chem..

[13] Li X., Du X., He J. (2010). Self-cleaning antireflective coatings assembled from peculiar mesoporous silica nanoparticles. Langmuir.

[14] Zhang F., Chen S., Dong L., Lei Y., Liu T., Yin Y. (2011). Preparation of superhydrophobic films on titanium as effective corrosion barriers. Appl. Surf. Sci..

[15] Xu L.Y., Zhu D.D., Lu X.M., Lu Q.H. (2015). Transparent, thermally and mechanically stable superhydrophobic coating prepared by an electrochemical template strategy. J. Mater. Chem. A.

[16] Laforte C., Beisswenger A. Icephobic Material Centrifuge Adhesion Test. Proceedings of the 11th International Workshop on Atmospheric Icing of Structures, IWAIS.

[17] Lai Y., Lin C., Huang J., Zhuang H., Sun L., Nguyen T. (2008). Markedly controllable adhesion of superhydrophobic spongelike nanostructure TiO_2_ films. Langmuir.

[18] Yu S., Guo Z., Liu W. (2015). Biomimetic transparent and superhydrophobic coatings: From nature and beyond nature. Chem. Commun..

[19] Pilotek S., Schmidt H.K. (2003). Wettability of microstructured hydrophobic sol-gel coatings. J. Sol-Gel Sci. Technol..

[20] Yang H., Deng Y. (2008). Preparation and physical properties of superhydrophobic papers. J. Colloid Interface Sci..

[21] Hejazi V., Sobolev K., Nosonovsky M. (2013). From superhydrophobicity to icephobicity: Forces and interaction analysis. Sci. Rep..

[22] Gohardani O. (2011). Impact of erosion testing aspects on current and future flight conditions. Prog. Aerosp. Sci..

